# Mapping Soluble Guanylyl Cyclase and Protein Disulfide Isomerase Regions of Interaction

**DOI:** 10.1371/journal.pone.0143523

**Published:** 2015-11-30

**Authors:** Erin J. Heckler, Vladyslav Kholodovych, Mohit Jain, Tong Liu, Hong Li, Annie Beuve

**Affiliations:** 1 Department of Pharmacology and Physiology and Neuroscience, New Jersey Medical School, Rutgers University, Newark, NJ, United States of America; 2 High Performance and Research Computing, OIRT, Rutgers University, New Brunswick, NJ, United States of America; 3 Department of Pharmacology, Robert Wood Johnson Medical School, Rutgers University, Piscataway, NJ, United States of America; 4 Proteomics Core, New Jersey Medical School, Rutgers University, Newark, NJ, United States of America; Maastricht University, NETHERLANDS

## Abstract

Soluble guanylyl cyclase (sGC) is a heterodimeric nitric oxide (NO) receptor that produces cyclic GMP. This signaling mechanism is a key component in the cardiovascular system. NO binds to heme in the β subunit and stimulates the catalytic conversion of GTP to cGMP several hundred fold. Several endogenous factors have been identified that modulate sGC function *in vitro* and *in vivo*. In previous work, we determined that protein disulfide isomerase (PDI) interacts with sGC in a redox-dependent manner *in vitro* and that PDI inhibited NO-stimulated activity in cells. To our knowledge, this was the first report of a physical interaction between sGC and a thiol-redox protein. To characterize this interaction between sGC and PDI, we first identified peptide linkages between sGC and PDI, using a lysine cross-linking reagent and recently developed mass spectrometry analysis. Together with Flag-immunoprecipitation using sGC domain deletions, wild-type (WT) and mutated PDI, regions of sGC involved in this interaction were identified. The observed data were further explored with computational modeling to gain insight into the interaction mechanism between sGC and oxidized PDI. Our results indicate that PDI interacts preferentially with the catalytic domain of sGC, thus providing a mechanism for PDI inhibition of sGC. A model in which PDI interacts with either the α or the β catalytic domain is proposed.

## Introduction

Soluble guanylyl cyclase (sGC) is the main receptor for nitric oxide (NO). The enzyme is composed of two subunits: α and β; where the β subunit contains an N-terminal heme, the site of NO binding. The catalytic site, formed by the obligate association of α and β subunits at the C-termini, converts GTP to cGMP. Upon NO binding, changes in enzyme conformation are transduced to the C-terminal catalytic domain, leading to hundred-fold stimulation of the catalytic activity.

Protein disulfide isomerase (PDI) has major roles in cellular oxidative protein folding and viral entry. These classical functions of PDI take place in the endoplasmic reticulum (ER) and at the cell surface, which are both oxidative environments. However, the cellular role of PDI is not limited to these functions. Laurindo and coworkers recently reported that PDI associates with NADPH oxidase [1]; subcellular fractionation found that PDI was present in the ER and cytosol which may suggest that PDI is involved in the organization of cytoskeleton and extracellular matrix via interaction with actin filaments that alter inter- and extra-cellular structures [2]. Others show that PDI localization may depend on oxygen tension [3–5]. Nonetheless, while the mechanism of domain interactions and the role of the two catalytic sites are well-defined between PDI and folding chaperone client proteins, the mechanism of PDI-target association outside the ER is less clear. Previously, we showed that PDI and sGC form a complex via thiol-disulfide exchange [6]. In addition, we had shown that sGC gets S-nitrosated [7] and this confirmed that sGC has reactive cysteines (Cys) that could form disulfide bonds. Moreover, this redox-dependent interaction between sGC and PDI is physiologically relevant, as we showed that PDI inhibits NO-stimulation of sGC in smooth muscle cells. A similar redox-dependent interaction was later found to occur between PDI and β-actin [2]. Thus, PDI-target complexes can occur in the reducing environment of cytosol. Understanding how domains of PDI and sGC associate could help to uncover the underlying mechanisms of protein interactions with thiol-oxidoreductases.

To determine which regions of sGC and PDI were involved in the interaction, we utilized a combined approach based on lysine cross-linking, mass spectrometry, and computational modeling. This allowed us to map contacts between the second active Cys site of PDI with peptides from the HNOX and catalytic domains of sGC.

## Materials and Methods

sGC from Axxora; all SDS-PAGE TGX gels, Bradford reagent, Coomassie Brilliant Blue, nitrocellulose from Biorad; dithiothreitol (DTT) from Denville Scientific; bis[sulfosuccinimidyl] suberate (BS3), immobilized TCEP resin from Thermo Scientific; Sequencing primers from Sigma Genosys; anti-sGC α, BSA, diamide, Flag-M2 affinity resin, iodacetamide, protease inhibitor cocktail from Sigma; PDI clone from Invitrogen; QuickChange II Kit from Stratagene; sGC α and β wild-type adenovirus amplified by Welgene. Anti-sGC α and β from Cayman; anti-Flag from Cell Signaling Technology; anti-PDI [RL-90] from AbCam.

### Molecular biology

PDI clone insert from Invitrogen in pCMV5-Sport 6 vector was sub-cloned out using restriction enzymes and Flag-tag added as previously [6]. Flag-tagged PDI-Ser-x-x-Ser2, replacement of the 2^nd^ active site Cys-x-x-Cys cysteines with serines, was done by site-directed mutagenesis using QuickChange II kit and the following primers: Cys-x-x-Ser–Forward: 5' GGTGTGGTCACTCCAAGCAGCTAGCC3'; Reverse: 5' GGCTAGCTGCTTGGAGTGACCACACC 3' and Ser-x-x-Ser–Fwd: 5'- GGAGTGGTCACTCCAAGCAGCTAGCC -3'; Rev: 5'- GGCTAGCTGCTTGGAGTGACCACTCC -3'. The insert was fully sequenced after each round of mutagenesis.

sGC α constructs were designed based on [8–10]. Three constructs: C-terminal deletion (αΔC; containing amino acids 1–449), N-terminal deletion (αΔN; containing amino acids 258–690), and catalytic domain alone (αCatDom; containing amino acids 466–690) were designed using the respective primers: N-term truncation:

Forward 5'GCAACGTCTAGAGCCGCCACCATGAAGAGCACCAAGCCTTCTCTG-3',

Reverse 5'-CACCCGGGATCCTCACTAATC-3';

C-term truncation Forward 5'-CTGCAGGTCGACTCTAGAGCC-3',

Reverse 5'-CACCCGGGATCCTCACTACGTCTTCTTCTTCTCCTCCTC-3';

catalytic domain:

Forward 5'-GCAACGTCTAGAGCCGCCACCATGCAAGGACAAATTGTGCAAGCC-3', reverse primer same as N-term truncation. The inserts were fully sequenced.

### Cell Culture

COS-7 cells were grown to 80–85% confluency before transient transfection in 100 mm dishes with 6 μg total DNA and Lipofectamine 2000 (Invitrogen) as per the manufacturer's instruction in Opti-MEM for four hours. After four hours, fresh complete media was added (DMEM, 5% FBS, Pen/Strep). COS-7 cells were transiently transfected with Flag-tagged PDI-WT or PDI-Ser-x-x-Ser2, in which both cysteines of the second active site were replaced (see above) for 24h, then infected with sGC adenovirus α or β WT at MOI of 1 or 10, respectively. After 24h post-infection, cells were harvested. For α constructs, COS-7 cells were co-transfected with 3 μg of α-WT, αΔC, αΔN, or αCatDom and 3 μg of PDI-WT-Flag or PDI-Ser-x-x-Ser2-Flag using Lipofectamine 2000 in Opti-MEM for four hours. After four hours fresh complete media was added (DMEM, 5% FBS, Pen/Strep). After 48 hours cells were harvested.

### Flag Immuno-precipitation

As previously [6], cells were harvested into lysis buffer: 50 mM TrisHCl, 150 mM NaCl with 10 μL protease inhibitor cocktail in 10 mL and sonicated at 30% output for 5 seconds. Lysates were harvested by centrifugation at 14000 RPM, 4°C for 10 minutes. Protein concentration determined by Bradford assay. 100 μg total protein added to 50 μL Flag-M2 affinity resin previously washed into lysis buffer. Western blots were analyzed using ImageJ (v. 1.45s) to determine densitometry of bands. The lysates (input) or elutes (bound) of sGC α-WT, αΔC, and αΔN were run on a 7.5% gel, transferred onto nitrocellulose and probed with Cayman anti-sGC α raised against the peptide containing amino acids 418–486. The αCatDom lysates and elutes were run on a 10% gel due to its much lower molecular weight, transferred onto PVDF, and probed with Sigma anti-sGC α raised against the peptide containing amino acids 673–690. The calculated molecular weight (M.W.) for each construct is as follows: 75 kDa α-WT, 48.5 kDa αΔN, 50.9 kDa αΔC, and 24.8 kDa αCatDom.

### Mass Spectrometry Sample Preparation

PDI (in pTRC-HisA, a gift from Dr. Colin Thorpe, University of Delaware) was purified as described previously [11]. sGC in 40 mM TEA, pH 7.4, containing 0.1 mM DTT, 1 mM EGTA, 1 mM EDTA and 25% glycerol was purchased from Axxora. Oxidized PDI and reduced sGC were incubated as described previously [6]. After the incubation period, the oxidized PDI and reduced sGC complex sample was alkylated with 40 mM iodacetamide at room temperature (22°C) for 1 hour. After alkylation, non-reducing Laemmli sample buffer was added and the sample was run on a non-reducing 7.5% TGX gel. The gel was stained with Coomassie and fixed in 5% methanol / 7% acetic acid overnight and washed into distilled water.

For cross-linking experiments, the preparation of the oxidized PDI/reduced sGC complex was the same as above. After incubation, a 50 molar excess of bis[sulfosuccinimidyl] suberate (BS3) was added and incubated for 30 minutes at room temperature (22°C) in the dark according to the manufacturer's instruction. The samples were then treated with 40 mM iodoacetamide in 50 mM Tris-HCl, pH 7.0 for one hour at room temperature in the dark to quench the reaction and alkylate the sample in preparation for mass spectrometry analysis. The cross-linked and alkylated samples were run on a non-reducing gel as above.

### Reversed Phase Liquid Chromatography Mass Spectrometry analysis (RPLC-MS)

The mass spectrometry data were obtained from an Orbitrap instrument (Rutgers Neuroproteomics Core Facility). Protein bands from Coomassie Brilliant Blue stained 1-D gels were picked for mass spectrometry analysis. The gel bands were diced into 1 mm^3^ pieces and washed with 30% acetonitrile (ACN) in 50 mM ammonium bicarbonate before DTT reduction and iodoacetamide alkylation. Trypsin was used for digestion at 37°C overnight. The resulting peptides were extracted with 30 μl of 1% trifluoroacetic acid followed by C18 ziptip desalting. Peptides were further fractionated by reversed phase liquid chromatography (RPLC) on an Ultimate 3000 LC system (Dionex, Sunnyvale, CA, USA) coupled with an Orbitrap Velos Pro Mass Spectrometer (Thermo Scientific) via a Thermo Scientific nano electrospray ionization source.

The mass spectrometer was operated in a Top 15 data dependent mode with automatic switching between MS and MS/MS. Source ionization parameters were as follows: spray voltage: 2.2 kV; capillary temperature: 275°C, s-lens: 50.0. Full scan MS mode (300–1650 m/z) was operated at a resolution of 60,000, automatic gain control (AGC) target; 1 × 106, maximum ion transfer time (IT): 500 ms. Ions selected for MS/MS were subjected to the following parameters: AGC: 5 × 104, IT 250 ms; 4.0 m/z isolation window; normalized collision energy 35.0 and dynamic exclusion: of 60.0 s.

### Mass spectrometric database search

The.RAW data files obtained from the mass spectrometer were converted to mascot generic format (.mgf) files using Proteome Discoverer (ThermoFisher, San Jose, CA). The mgf files were searched against custom protein databases (human sGC α GCYA3 and β GCYB1 and human PDI P4HB) using the MassMatrix search engine (www.massmatrix.net). The custom FASTA format protein databases were composed of target protein sequences and decoy protein sequences. Decoy protein sequences were reversed or randomized sequence of sGC. All spectra that were not determined as singly charged were searched as both doubly and triply charged ions. Peptide sequences with a length from 6 to 50 amino acid (AA) residues, missed cleavage sites of up to 4, and charges of +1, +2, and +3 were searched. For spectra with multiple matches, the highest scoring match was used.

The MassMatrix search engine employs a probabilistic scoring model and fast database searching algorithm for identification of disulfide bonds from proteins and peptides as explained in detail here [12,13]. In brief, MassMatrix initially digests the protein sequence(s) *in silico* according to the enzyme or cleavage sites specified, in this case trypsin. The resulting peptide sequences are fragmented *in silico* and then matched against the MS/MS data. Three scores (score, pp, and pp2 values) are calculated for each potential match; matches below the critical thresholds are discarded. The pp score is used to evaluate whether the number of matched product ions in an experimental spectrum could be a random occurrence. The pp2 score evaluates whether the total abundance of matched product ions in the experimental spectrum could be a random occurrence. The protein scores reflect the significance of protein matches and are used to differentiate true protein matches from random matches.

### Computational Modeling

The catalytic domain of sGC (PDB 4NI2) and structure of oxidized PDI (PDB 4EL1) were taken from a protein databank and then manually refined in Molecular Operating Environment (MOE 2013.0801) [14]. Protein crystal structures were stripped off salt and solvent molecules and in case of multiple chains only one instance of each protein was retained. The remaining structures were processed through the “Structure Preparation” subroutine from MOE and all broken and missing residues were repaired and protonated at pH 7. C-Terminal tails of sGC were modeled in extended conformation by adding missing residues G663-D690 (α subunit) and T609-D619 (β subunit) to the available X-ray structure of the catalytic domain of sGC (PDB 4NI2, α 471–662, β 411–608). Amino acid sequences of the human α and β subunits of sGC were obtained from UniProt portal, access codes Q02108 and Q02153, respectively [15].

After minimization in MOE, sGC extended models were subjected for further refinement with molecular dynamics (MD) with the Amber 14 molecular dynamics package [16].

Each sGC model was solvated with water and neutralized with sodium ions. Molecular dynamics simulations were performed for 4 different models over 100ns each to capture the random re-organization of the added tail residues. After initial system minimization and equilibration for 1ns, an isothermal–isobaric (NPT) ensemble with Langevin dynamics was used throughout the MD simulation with restart checkpoints every 1ns. The average positions of the added residues were calculated over the last 50ns of the MD simulations from Amber trajectory files, minimized with a sander program from the Amber 14 and used in this study.

A rigid docking between sGC and PDI were carried out on GRAMM-X Protein-Protein Docking Web Server v.1.2.0 (http://vakser.compbio.ku.edu/resources/gramm/grammx/) [17,18]. sGC was set as a receptor and PDI as its ligand and for a potential ligand-receptor docking interface several regions around pairs of cross-linked residues observed after mass-spec analysis were selected: sGC α Lys672 and PDI Lys409; sGC β Lys615 and PDI Lys370. A hundred GRAMM-X generated docking complexes were transferred back to MOE for visual inspection and further refinement. Only docking models with the distances between selected lysine pairs in the range of 15 Å or less, based on the length of the Lys side chains plus the approximate length of the cross-linking reagent BS3, were considered to be acceptable. These docking models were subjected for a short 10ns MD refinement with the same protocol as described earlier for sGC extended models.

## Results

### Determination of PDI and sGC interacting regions

**Fig 1:**
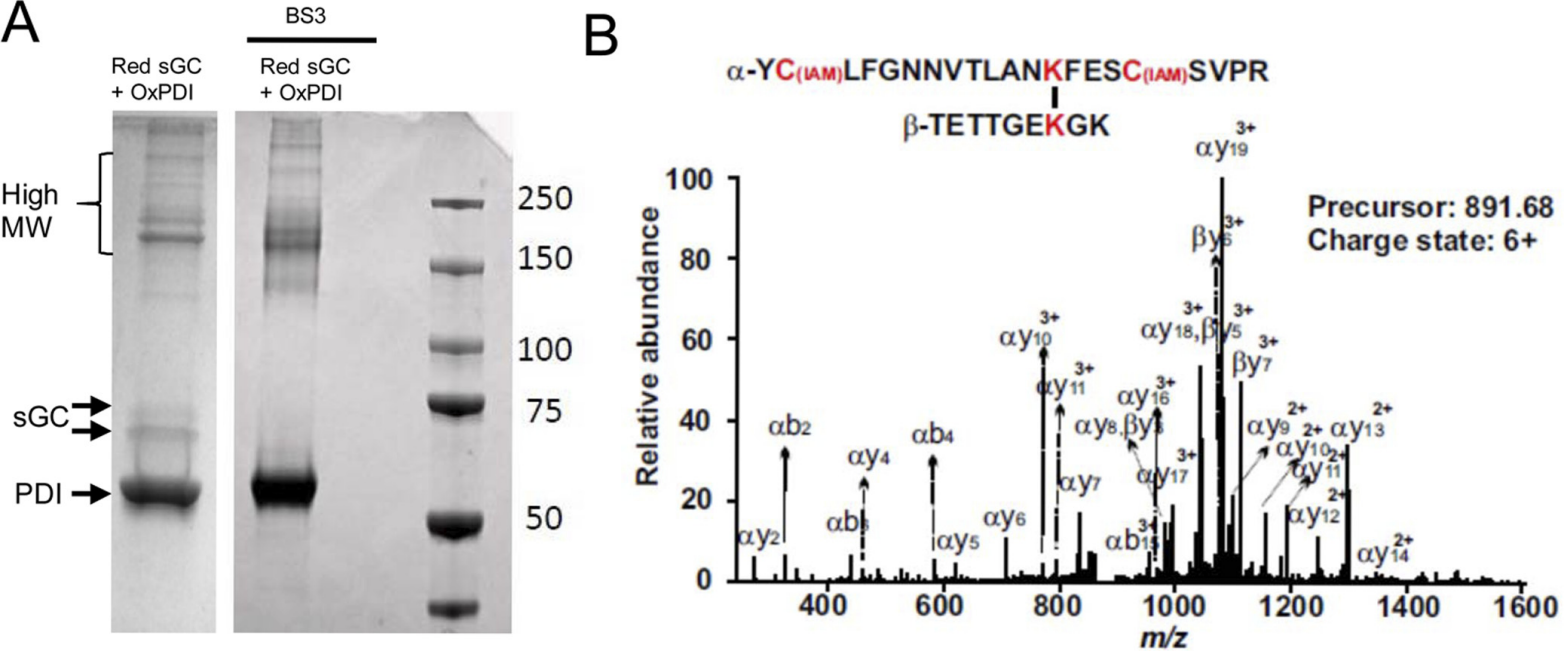
BS3 cross-linking of reduced sGC and oxidized PDI and mass spectrometry analysis. Reduced sGC and oxidized PDI complex were incubated in the absence (left panel) or presence (right panel) of the lysine cross-linking reagent, BS3, then alkylated and run on 7.5% TGX gel under non-reducing conditions. Gel was stained with Coomassie blue. Representative MS/MS spectrum of a cross-linked peptide. A 6+ precursor with the m/z of 891.68 corresponds to the cross-linked peptides YCLFGNNVTLANKFESCSVPR and TETTGEKGK with K606 from sGC α subunit and K559 from sGC β subunit cross-linked. All the b- and y- series of fragments and cross-linked fragments confirmed the peptide sequence. The fragments α y9 - α y14, α y16 – α y19, β y3- β y7 indicate that α K606 and β K559 are crosslinked.

We showed previously that the interaction between sGC and PDI takes place through a mixed disulfide exchange and is mostly transient [6]. This interaction was only observed between reduced sGC and oxidized PDI. PDI is a four domain protein with homologous N- and C-terminal WCGHC active sites, where the two redox active Cys are separated by Gly and His. When PDI is oxidized, each active site Cys-x-x-Cys forms a disulfide bond. To investigate the regions of interaction between PDI and sGC, reduced sGC and oxidized PDI were incubated in the absence or presence of the lysine cross-linking reagent, BS3. This reagent was previously used by the Montfort group to determine sGC dimer and domain interactions on a truncated construct (without the catalytic domains) [9]. The samples were next alkylated then run on an SDS-PAGE gel (Fig 1A). The BS3 linkage, as expected, increased the density of the high Molecular Weight (MW) bands and reduces the sGC α and β subunits/monomers. This switch in the band migration indicates that the BS3 reagent effectively crossed-linked PDI-sGC.

### MS identification of cross linked peptides between sGC and PDI

**Fig 2:**
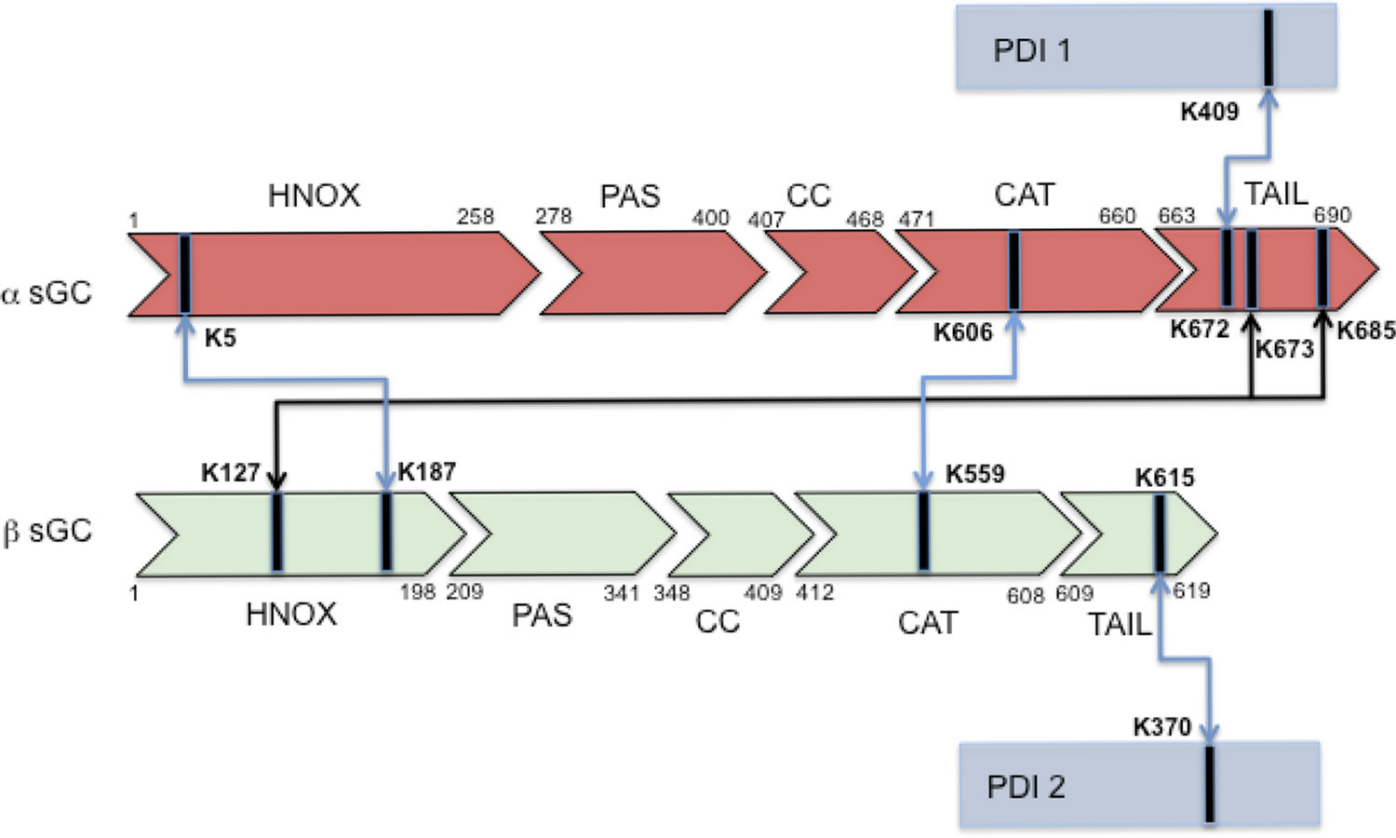
Scheme of lysine interactions between sGC and PDI. For clarity, PDI is represented twice as blue box 1 and 2, above and below α and β subunits of sGC. α and β are represented as light red and light green block arrows, respectively. The respective HNOX, PAS, coiled-coil (CC), catalytic domain (CAT) and C-terminal tail are indicated with the corresponding residue numbers (human sequence).

The pattern of BS3-Lys crosslinks between sGC α/β and PDI were analyzed from tandem MS data using the MassMatrix search engine as described in methods with BS3 linkage as the modification. Fig 1B shows a representative cross-linked peptide MS/MS spectrum between α and β subunits in the catalytic domain α Lys606 with β Lys559. We found that PDI molecules interact with sGC through the region that contains the second Cys-x-x-Cys active site and that sGC is crosslinked at both C-terminal catalytic domain of α and β subunits via the following lysines linkage: PDI Lys409 to sGC α Lys672 and PDI Lys370 to sGC β Lys615. Additionally, sGC inter-subunit cross-links were identified: α K5 with β Lys187, α Lys606 with β Lys559, α Lys673 with β K127, and α Lys685 with β Lys127 (Fig 2 and Table 1).

**Table 1:** Identified Cross-Linked Lysines between sGC (inter and intra subunits) and PDI.

sGC α	sGC β	PDI
K5—->	<—-K187	
K606—->	<—-K559	
K672—->		<—-K409
K673—->	<—-K127	
K685—->	<—-K127	
	K615—->	<—-K370

### PDI interacts preferentially with sGC α

**Fig 3:**
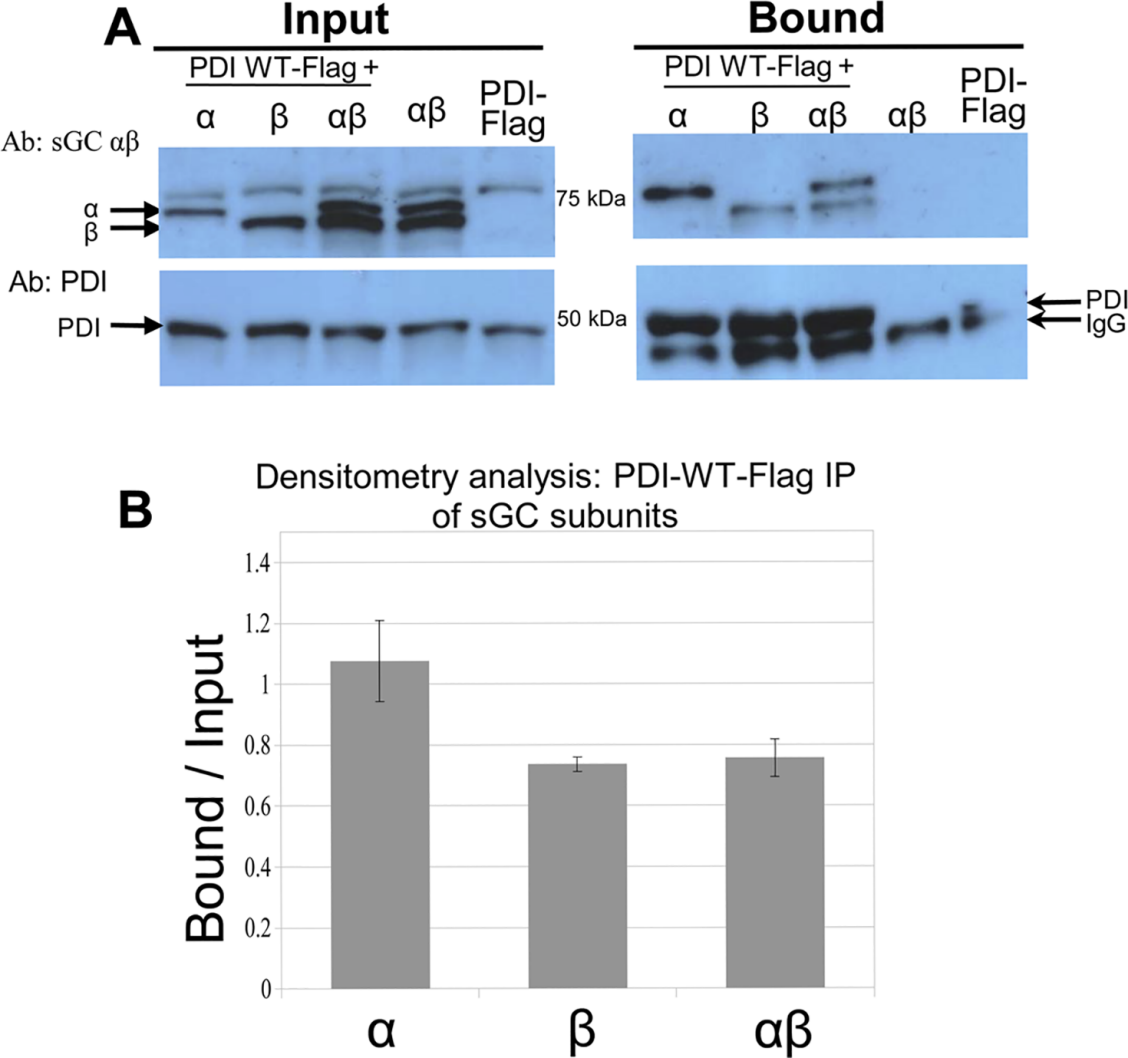
Flag immunoprecipitation of α and/or β sGC by Flag-tagged PDI-WT. **A.** Representative Western blots of α, β and Flag-PDI expressed in COS-7 cells (input right panel) and precipitated by PDI-Flag (bound, right panel). COS-7 cells were transiently transfected with Flag-tagged PDI-WT and infected with sGC α and/or β WT adenoviruses 24 hours post-transfection. Immunoprecipitation of lysates was performed using anti-Flag affinity resin as described in Material and Methods. Blots were probed with anti-α and β (top panels) and PDI (bottom panels). **B.** Densitometry analysis of the blots. Band analysis of bound vs. input with ImageJ of three separate experiments; sGC bound over input: α (1.08 ± 0.13), β (0.74 ± 0.02), and αβ (0.76 ± 0.06).

The MassMatrix and cross-linking mass spectrometry results showed that the catalytic domains of both subunits could interact with PDI. To determine whether PDI might have a stronger affinity for a particular sGC subunit, i.e. α vs. β, and whether the heterodimer is required for interaction, WT sGC α and β, separately or together were co-expressed with Flag-tagged WT PDI (PDI-WT-Flag) in COS-7 cells, which have no detectable sGC. PDI-Flag immunoprecipitation (Flag-IP, Fig 3) indicated that both α and β, separately or as a heterodimer were pulled down by PDI, yet the signal was stronger with α subunit alone compared to sGC β alone or α+β, as measured by densitometry analysis (1.08 ± 0.13 vs. 0.74 ± 0.02, 0.76 ± 0.06). This result suggests that PDI interaction with sGC does not require the heterodimer conformation and that the α subunit more readily interacts with PDI in an exogenous expression system. Interestingly, the decreased pull-down by PDI of α when co-expressed with β (compared with α subunit expressed alone) while PDI pull-down of β is similar when expressed alone or with α, suggests that there is a competition between PDI binding to sGC subunits and to each other.

### PDI interacts more strongly with the sGC α catalytic domain

**Fig 4:**
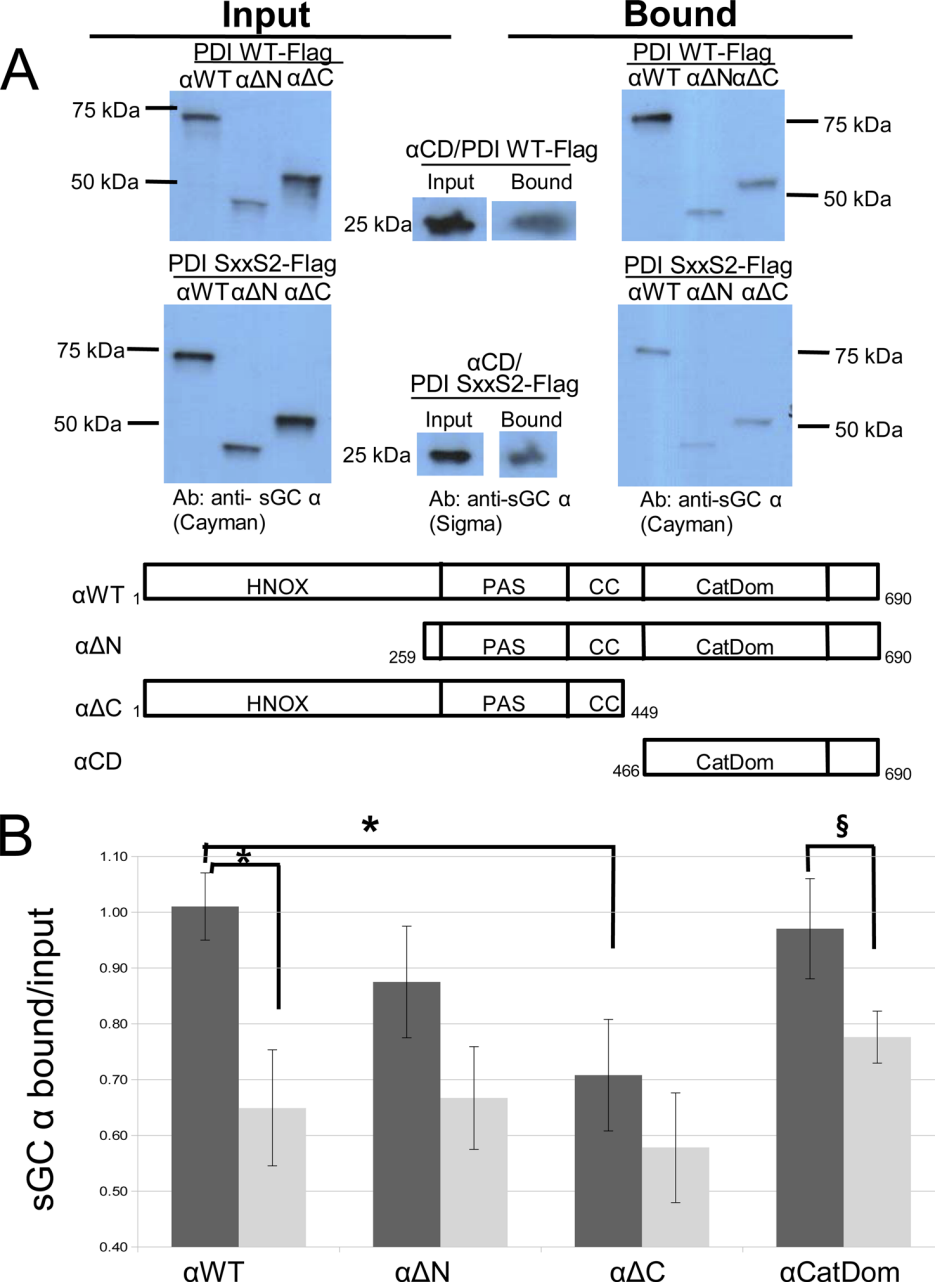
α sGC domains immunoprecipitation by Flag-tagged PDI-WT and Flag-PDI Ser-x-x-Ser2. **A.** Representative Western blots of lysates (input) and immunoprecipitates (bound) using PDI-Flag and PDI-Flag mutant. COS-7 cells were transiently co-transfected with Flag-tagged PDI-WT or PDI Ser-x-x-Ser2 (PDI SxxS2) and sGC α domain constructs: αWT: full-length, αΔN: N-terminal truncation, αΔC: C-terminal truncation, α-CD: catalytic domain. Immunoprecipitation of lysates was performed using anti-Flag affinity resin as described in Material and Methods. Blots were probed with anti-sGC α Cayman and anti-sGC α Sigma. Bottom panel showing α constructs with domain boundaries labeled. **B.** Densitometry analysis of bound vs. input with ImageJ of three to five separate experiments. Black bars: PDI WT; gray bars: PDI SxxS2. Statistical analysis using Student's t-Test: α with PDI WT and α with PDI SxxS2, p = 0.01*; α with PDI WT and αΔC with PDI WT, p = 0.02*; and α-CatDom with PDI WT and α-CatDom with PDI SxxS2 = 0.06§.

To determine which domains of the sGC α subunit are involved in interaction with PDI, we made truncated constructs in pCMV5 expression plasmids as follows: the N-terminal deletion (αΔN), C-terminal deletion (αΔC), and catalytic domain alone (αCatDom) [8–10]. These constructs were co-expressed with a Flag-tagged PDI-WT or with a Flag-tagged PDI mutated in the second active site Cys^397^-x-x-Cys^400^, which is responsible for mixed-disulfide exchange with sGC (PDI Flag-Ser-x-x-Ser2, both Cys mutated to Ser). The results are shown in Fig 4A. PDI-Flag IP of αΔN and αCatDom were not significantly changed compared to α WT. However, αΔC (no catalytic domain) associated with PDI significantly less than full-length α WT (0.71±0.10 vs. 1.01±0.06: n = 3–4 densitometry analysis, Fig 4B). An interaction of each α domain construct, including α WT, with mutated PDI-Ser-x-x-Ser2 was impaired (Fig 4A). Yet, only the catalytic domain of α (αCatDom, 460-690aa) and α WT were significantly affected in their interaction with PDI-Ser-x-x-Ser2 compared to PDI WT (Fig 4B). The results confirmed that association between sGC and PDI involves PDI second active site interaction with the α catalytic domain (αCatDom, αCD).

### Computational model of the PDI-sGC interaction

**Fig 5:**
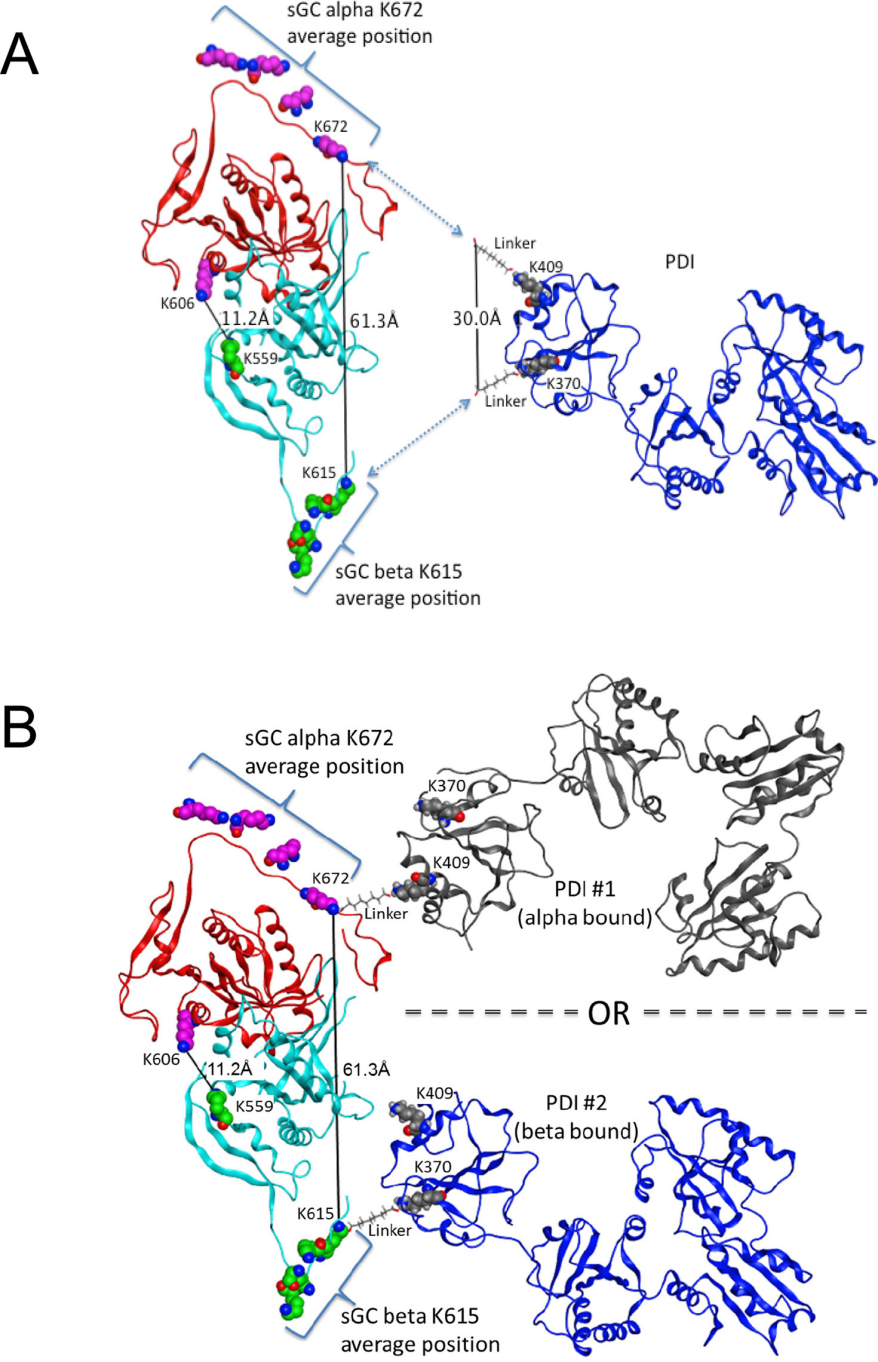
Proposed computational model of the sGC-PDI interaction Representative models of sGC and PDI interaction with possible distances between cross-linked residues are shown. Color-coding: α subunit of sGC is depicted as red ribbon, β subunit of sGC is light blue ribbon, PDI are dark blue and black ribbons. Average positions from four 100ns MD simulations of sGC residues α K672 (magenta) and β K615 (green) and the corresponding interacting residues of PDI K370 and K409, and the pair of cross-linked residues α K606 and β K559 of sGC are shown as van der Waals surfaces. **A.** The possible closest distance between sGC residues α K672 and β K615 is 61.3Å, which is twice more than the required distance to form a contact with both K370 and K409 residues of one molecule of PDI with linkers (30Å). **B.** One molecule of PDI interacts with one at a time molecule of sGC. PDI #1 interacts with sGC via α catalytic domain or PDI #2 interacts with sGC via β catalytic domain.

There is no full-length crystal structure or model of sGC available. In addition, the most recent PDB entry of the catalytic domain of sGC (PDB: 4NI2) [19] has both α and β subunits truncated at C-termini. Coincidentally, these truncated regions are where the cross-links between sGC (at α Lys672 and β Lys615) and PDI occur. Thus, we employed computational modeling to extend the available crystal structure of the sGC catalytic domain and then used it for docking with PDI molecules. First, the missing C-termini amino acid residues, α G663-D690 and β T609-D619 were added to the available X-ray structure of the catalytic domain of sGC (PDB 4NI2, α 471–662, β 411–608). Because the structural organization of these flexible tails are not known, we used extended conformations of peptides and then performed molecular dynamics (MD) simulations to capture the random re-organization of the added residues. Four independent MD models were produced after 100ns simulation. The visual analysis of the models revealed that the possible closest distance between sGC residues α Lys672 and β Lys615 is 61.3Å, which is twice more than the required distance to form a contact with both Lys370 and Lys409 residues of one molecule of PDI with linkers (30Å), Fig 5A. Therefore, it is not possible for one molecule of PDI to interact with both α and β subunits of sGC at the same time. Hence we posit that one PDI molecule interacts with the α subunit *or* the β subunit of sGC in a mutually exclusive manner and performed protein-protein docking on the GRAMM-X website with a followed-up short MD refinement. The two exclusive interactions are shown in Fig 5B. The only other crosslink that was observed in the modeling region of the sGC catalytic domain after mass-spec analysis was an inter-subunit contact between α Lys606 and β Lys559. The distance between sGC α Lys606 and β Lys559 in our model was 11.2Å which is exactly the length of the BS3 linker (11.3Å). We used this distance as an internal control of the models after MD simulation and docking. Any model that violates this distance was discarded.

## Discussion

We previously reported on the redox dependent interaction between sGC and PDI and inhibition of NO-stimulated sGC activity by PDI [6]. Herein, using mass spectrometry combined with chemical cross-linking, the regions of sGC and PDI involved in their interaction were mapped. These findings were validated by Flag-immunoprecipitation using truncated forms of α sGC where Flag-tagged PDI WT preferentially interacts with the α subunit catalytic domain. Using a computational strategy it was possible to dock PDI WT with the sGC α/β catalytic domains at two sites. Analyzing this docking model in the context of sGC function suggests a mechanism for PDI inhibition of sGC NO-stimulation that is discussed further below.

BS3 is a lysine cross-linking reagent used in this work to delineate PDI and sGC regions in combination with MS. This same reagent was used in work by the Montfort group to study sGC conformational changes in the N-terminal half of sGC (e.g. without the catalytic domain) following stimulation of sGC activity [9]. Using full-length reduced sGC and full-length oxidized PDI, we identified BS3 cross-linked peptides that included the region containing the PDI second redox active Cys-x-x-Cys site (PDI Lys370 and Lys409), and the sGC C-terminal catalytic domains (β Lys615 and α Lys672). This was confirmed by combining immuno-precipitation (IP) domain deletion studies showing that PDI WT could still associate with the sGC α subunit missing the N-terminus (αΔN, 258-690aa) and with the catalytic domain alone (αCatDom 466-690aa), while the association was significantly decreased when the catalytic domain was missing (α C-terminal deletion, αΔC 1-449aa). However the interaction was not completely abolished, therefore it is likely that there is more than one site where sGC and PDI interact. Redox impaired PDI-Ser-x-x-Ser2 had decreased association with α WT and all domain constructs, but it was significant only with α WT and the catalytic domain construct. In addition to supporting our finding that PDI main interaction is with the αCatDom, it also confirms observations from our previous work that the interaction is thiol redox-dependent, unlike other complexes (sGC-Hsp90 and sGC-p53) found by others, see below [20,21]. This may indicate that there is a cellular function for the sGC-PDI interaction and that function depends on the cellular redox state.

Of note, the Flag IP experiments indicated that the interaction PDI-sGC α can occur in the absence of β, i.e. in the absence of heterodimer. The ability of one sGC subunit to interact as a monomer “outside” the heterodimer was recently shown for the apo-form of sGC β with Hsp90 [20] and for sGC α subunit with the tumor suppressor p53 [21]. We confirmed by co-IP that all α sGC constructs that associate with PDI can associate with the β subunit and that the α C-terminal domain that interacts with PDI is also requires for interaction with the β subunit (S1 Fig). Together with the observation that the α subunit in a complex with β (heterodimer (α/β) is less pulled down by PDI than the α subunit expressed alone (and β is similarly pulled down as a single subunit or as an heterodimer, Fig 3), it is possible that PDI, to a certain extent, “competes” or interferes with sGC α catalytic domain for binding to the β subunit.

Using the catalytic domain of sGC and full-length oxidized PDI crystal structures, we conducted a computational docking study based on the MS and IP data. The first observation was that oxidized PDI has to interact separately with the catalytic C-terminal part of α and of β due to the distance limitation required for cross-linking. This is consistent with our native gel results (Fig 1A) showing that the cross-linked bands migrate around 200 kDa, suggesting a complex between one PDI molecule (50 kDa) and one sGC molecule (150 kDa).The linkage study showed that Lys606 of α and Lys559 of β are linked when oxidized PDI and reduced sGC are mixed. The computer modeling confirmed that these two lysines could be connected by an 11Å BS3 reagent. In a recent hydrogen/deuterium exchange mass spectrometry (HDX-MS), Underbakke et al. [22] identified the Lys606-containing α region as buried (very slow exchange) when the catalytic domain is mixed with full-length sGC or with the β HNOX domain. This suggests that this region is relevant for inhibition of catalytic activity by HNOX domain, and was the confirmation of a previous biochemical study showing an inhibition in trans of sGC activity by addition of β HNOX [23]. In our current study, we speculate that PDI inhibits sGC activity by a similar mechanism. In the other HDX-MS study of the NO-induced conformational change[24], both peptides containing α Lys606 and β Lys559 are regions where the exchange rate is among the lowest in the presence of NO, thus suggesting a potential “collapse” of the catalytic site. The cross-linked α Lys606-β Lys559 peptide, whether or not it is induced by oxPDI, reflects this contracted conformation of the sGC catalytic pocket and support the idea that these 2 regions are key for catalytic activity (S2 Fig). The other identified cross-linked peptides, α Lys5- β Lys187, α Lys673-β Lys127 and α Lys685-β Lys127 cannot be included in our computer model because they belong to different domains of sGC, for which we do not have the full-length 3D structure Interestingly, these 3 cross-linked peptides involve lysines (Lys127 or Lys187) from the HNOX domain of β, which as the site of NO binding, is the key domain for modulation of sGC activity. A recent study using single-particle EM [25], suggests that the EM density map could be represented in a “bent conformation” in which the β HNOX domain directly interacts with the α1 catalytic domain; thus we propose that the α Lys673-β Lys127 and α Lys685-β Lys127 linked peptides reflect this bent conformation.

Several groups, including ours, have used different approaches (HDX-MS, FRET, Lysine linkage, single-particle EM, mutational analysis, [8,9,20,22,25–28]) and different constructs in an attempt to understand how the various domains of sGC interact, and how the NO signal is propagated from the heme receptor domain to the catalytic effector domain. The work presented here brings the additional dimension of a thiol-redox dependent association and modulation of sGC. Together with our previous report that PDI inhibits NO-stimulated sGC activity *in vitro*, our data and computational modeling studies lead us to propose that PDI interacts with the α catalytic domain to prevent NO-stimulation of sGC in a similar way to how the HNOX domain achieves auto-inhibition under basal conditions.

## Supporting Information

S1 Fig All α sGC domain constructs that IP with PDI also IP with the β subunit. COS-7 cells were co-transfected with each α construct and β WT. Anti-sGC β was used to co-immunoprecipitate α WT and the three α domain constructs αΔN (lacking the N-terminal domain), αΔC (lacking the C-terminal domain) and α catalytic domain (α CD that expresses only the catalytic domain). Anti-β antibodies pull down the α WT construct, αΔN (black arrows IP panel) and α CD (right panel) but not the α C-terminal deletion construct, as confirmed by the unbound fraction that shows a strong signal for αΔC (e.g. no pull-down). These data suggest that the catalytic C-term domain of α is required for heterodimerization and for interaction with PDI. Control for specificity of IP was IgG. The bracket indicates IgG signals. Two different antibodies have to be used to detect α construct (from Cayman and Sigma).
(PDF)

S2 Fig Modeling of the αK606-βK559 cross-link in the sGC catalytic domain. Catalytic Asp 530 and Asp 486 are shown as blue balls and sticks. An active site Asp486 is a part of an antiparallel beta-sheet formation in the α subunit (Val480-Val488; Ile571-Gly580; Lys615-Ser619). A beta-sheet is shown as yellow ribbons. Lys606 is a member of the alpha helix shown as red ribbons (Gly598-Ser619). The helix is adjacent to the beta-sheet and forms a complex hydrogen bond networking with it. Interactions between amino acid residues in alpha helix and beta-sheet stabilize the active site of sGC, especially the position of Asp486, which is directly involved in hydrogen bond networking. Thus a formation of the BS3 link between the α subunit Lys606 and the β subunit Lys559 may reflect an inhibitory conformation in which the alpha helix get closer to the β subunit and modify the catalytic pocket.
(PDF)
