# Supplementary material for: Mapping Soluble Guanylyl Cyclase and Protein Disulfide Isomerase Regions of Interaction
Source: PLoS One. 2015 Nov 30;10(11):e0143523. doi: 10.1371/journal.pone.0143523 (PMC4664405; doi:10.1371/journal.pone.0143523)
Supplement: S1 Fig — COS-7 cells were co-transfected with each α construct and β WT. Anti-sGC β was used to co-immunoprecipitate α WT and the three α domain constructs αΔN (lacking the N-terminal domain), αΔC (lacking the C-terminal domain) and α catalytic domain (α CD that expresses only the catalytic domain). Anti-β antibodies pull down the α WT construct, αΔN (black arrows IP panel) and α CD (right panel) but not the α C-terminal deletion construct, as confirmed by the unbound fraction that shows a strong signal for αΔC (e.g. no pull-down). These data suggest that the catalytic C-term domain of α is required for heterodimerization and for interaction with PDI. Control for specificity of IP was IgG. The bracket indicates IgG signals. Two different antibodies have to be used to detect α construct (from Cayman and Sigma). (PDF) [file pone.0143523.s001.pdf]

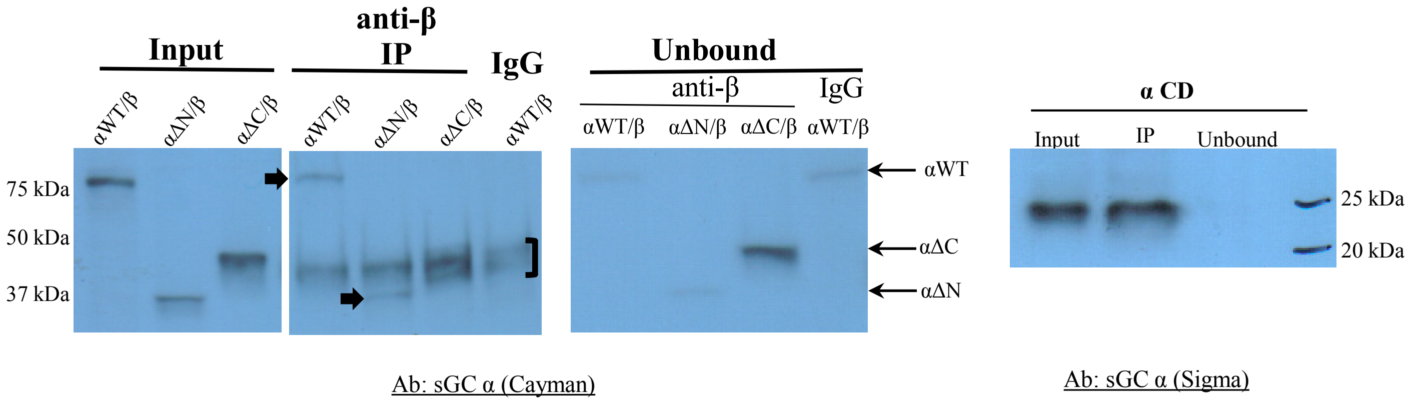

S1 Fig : All α sGC domain constructs that IP with PDI also IP with the β subunit.

COS-7 cells were co-transfected with each α construct and β WT. Anti-sGC β was used to co-immunoprecipitate α WT and the three α domain constructs αΔN (lacking the N-terminal domain), αΔC (lacking the C-terminal domain) and α catalytic domain (α CD that expresses only the catalytic domain). Anti-β antibodies pull down the α WT construct, αΔN (black arrows IP panel) and α CD (right panel) but not the α C-terminal deletion construct, as confirmed by the unbound fraction that shows a strong signal for αΔC (e.g. no pull-down). These data suggest that the catalytic C-term domain of α is required for heterodimerization and for interaction with PDI. Control for specificity of IP was IgG. The bracket indicates IgG signals. Two different antibodies have to be used to detect α construct (from Cayman and Sigma).
