# Supplementary material for: Mapping Soluble Guanylyl Cyclase and Protein Disulfide Isomerase Regions of Interaction
Source: PLoS One. 2015 Nov 30;10(11):e0143523. doi: 10.1371/journal.pone.0143523 (PMC4664405; doi:10.1371/journal.pone.0143523)
Supplement: S2 Fig — Catalytic Asp 530 and Asp 486 are shown as blue balls and sticks. An active site Asp486 is a part of an antiparallel beta-sheet formation in the α subunit (Val480-Val488; Ile571-Gly580; Lys615-Ser619). A beta-sheet is shown as yellow ribbons. Lys606 is a member of the alpha helix shown as red ribbons (Gly598-Ser619). The helix is adjacent to the beta-sheet and forms a complex hydrogen bond networking with it. Interactions between amino acid residues in alpha helix and beta-sheet stabilize the active site of sGC, especially the position of Asp486, which is directly involved in hydrogen bond networking. Thus a formation of the BS3 link between the α subunit Lys606 and the β subunit Lys559 may reflect an inhibitory conformation in which the alpha helix get closer to the β subunit and modify the catalytic pocket. (PDF) [file pone.0143523.s002.pdf]

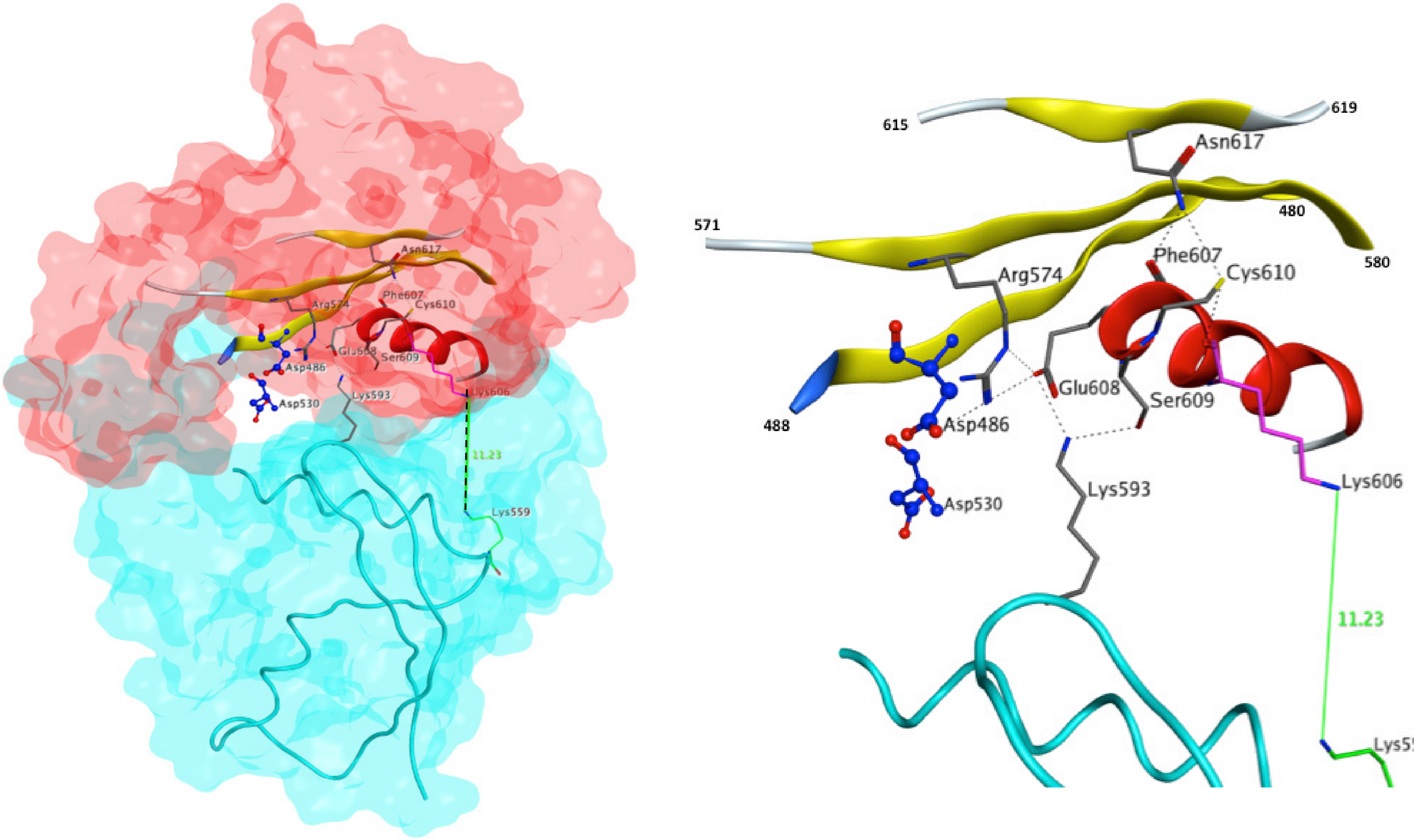

S2 Fig. Modeling of the αK606-βK559 cross-link in the sGC catalytic domain

Catalytic Asp 530 and Asp 486 are shown as blue balls and sticks. An active site Asp486 is a part of an antiparallel beta-sheet formation in the α subunit (Val480-Val488; Ile571-Gly580; Lys615-Ser619). A beta-sheet is shown as yellow ribbons. Lys606 is a member of the alpha helix shown as red ribbons (Gly598-Ser619). The helix is adjacent to the beta-sheet and forms a complex hydrogen bond networking with it. Interactions between amino acid residues in alpha helix and beta-sheet stabilize the active site of sGC, especially the position of Asp486 which is directly involved in hydrogen bond networking. Thus a formation of the BS3 link between the α subunit Lys606 and the β subunit Lys559 may reflect an inhibitory conformation in which the alpha helix get closer to the β subunit and modify the catalytic pocket.
